# Skull Base Csf- Leak Closure with Autologous Fibrin Sealant

**DOI:** 10.22038/ijorl.2020.42520.2385

**Published:** 2021-01

**Authors:** Salvatore Poma, Domenico-Michele Modica, Gianfranco Mattina, Salvatore Gallina, Alfio-Alfonso Azzolina, Giuseppe Mario-Galfano

**Affiliations:** 1 *Otorhinolaryngology Unit,* *Villa Sofia-Cervello Hospital, Palermo, Italy.*; 2 *Otorhinolaryngology Unit, Department of Bi. N. D., University of Palermo, Palermo, Italy.*; 3 *Otolaryngology Unit, Cannizzaro Hospital, Catania, Italy.*

**Keywords:** Autologous fibrin sealant, Cerebrospinal Fluid Leak, Haemostatic, Skull base, Vivostat®, Graft

## Abstract

**Introduction:**

There are many fibrin-derived sealants used as topical haemostatic agents in many surgical procedures. Fibrin sealants are usually non-autologous derivatives or animal derivatives, with the exception of Vivostat®, an autologous fibrin sealant derived from patients own blood.

**Materials and Methods:**

We present our experience on the use of Vivostat® in skull base closures in 20 patients operated at the Otorhinolaryngology Unit of the Hospital Ospedali Riuniti Villa Sofia - Cervello of Palermo. All postoperative patients were placed in an anti-trendeleburg position for 48 hours. After removal of the nasal swabs we did not find any rhinorrhea and we checked the tightness of the skull base defect with computed tomography.

**Results:**

On a total of the 20 patients (10 post-traumatic and 10 with iatrogenic leaks), 9 out of 10 post-traumatic cases had a leak in the border area between the anterior and posterior portion of the ethmoid, while 1 patient out of 10 post-traumatic cases had a leak at the level of the sella. In all 20 patients, we repaired skull base defects by fixing grafting materials with Vivostat®. We have not had any complications. Vivostat® is a useful product in skull base repair and safe for the patients.

**Conclusion:**

Vivostat® has been used as a sealant on body tissues with greater elasticity and more resistant allowing better and safer wound repair, especially in skull base surgery. In particular, its immediate polymerisation is very useful for an evaluation of the mechanical sealants in the closure of the skull base cerebrospinal fluid leak.

## Introduction

Communication via fistula between the skull base and the nasal cavities determines the cerebrospinal fluid (CSF) rhinorrhea, and usually consequent to various situations such as facial trauma, iatrogenic damage and neoplasms. Physical examination, endoscopy, radiologic exams and biomarker technology are used for correct diagnosis. It is very important to make rapid diagnosis and a treatment plan because the most important risk of CSF rhinorrhea is meningitis. It is very important to decide on surgical or non-surgical treatment, taking into account the etiology, time and size of the fistula. A lot of fibrin sealants are used in many different fields of surgery to control bleeding, provide a rapid firm seal in seconds and fixing grafting material to accelerate tissue restoration and repair. Fibrin sealants are usually non-autologous human or animal derivatives, whereas Vivostat^®^ autologous fibrin derives from the fibrinogen and thrombin of the patient. The purpose of our work is to describe our results in skull base CSF leaks closure, after various types of skull base procedures using Vivostat^®^.

## Materials and Methods

We performed a retrospective study on a cohort of 20 patients; 14 males and 6 females, of average age 49.9 years old. These patients were treated endoscopically transnasally, for CSF-leak in the Otolaryngology Unit at Palermo Villa Sofia-Cervello Hospital from January 2016 to December 2018. All patients remained for 24h intubated under pharmacological coma after surgery, in the intensive care unit (ICU) in the anti-trendelenburg decubitus position. All patients underwent a control CT scans at 48 hours in a. Merocel nasal packing was removed after 72 hours in all patients and control endoscopy was performed. All patients underwent after 1 month a CT scan and an endoscopic control. We evaluated the presence of any residual leaks with CT scan and endoscopy and recorded our results. In follow-up all patients underwent CT scan and endoscopy after 1 month.

Vivostat^®^ is an instantly system for preparation of autologous fibrin sealant and haemostasis in surgery field. It is a reproducible preparation fully automated that prepares 5-6,5 ml of autologous fibrin sealant from 120 ml of the patient's own blood in 23 min. The Vivostat® concentration of fibrin and the volume of sealant are stable and it’s possible to use it until up to 8 hours from its preparation without losing its proprieties keeping it at controlled temperature. 

This new system is fully automatic and is composed by three elements: processor unit, applicartor unit and disposable set. The technology guarantees a rapid polymerization. 

The mechanism for activating the Vivostat^® ^system consists in the non-enzymatic polymerization triggered by the change in ph which causes the rapid activation of the coagulation process. 

The differences with respect to the other systems consists in eliminating the risk of human or bovine contamination (Parvovirus B19, HIV, HBV, HCV) because it is autologous derivative. The sealant with a special endoscopic-device can be applied pin-pointed or sprayed uniformly with quick polymerising action. 

Vivostat^® ^system is an automatic autologous fibrin preparation procedure and the costs are similar to those of commercial glues already in use. All the patients provided an informed consent to the surgical procedure and our institutional review board approved this study.

## Results

In all 20 patients, we repaired skull base defects by fixing grafting materials (fascia lata, adipose tissue, Hadad flap) with Vivostat^®^. Of the 20 patients (Table. 1), 10 came to our attention as a result of post-traumatic injury with liquorrea, and 10 patients with iatrogenic leaks. 

**Fig 1:**
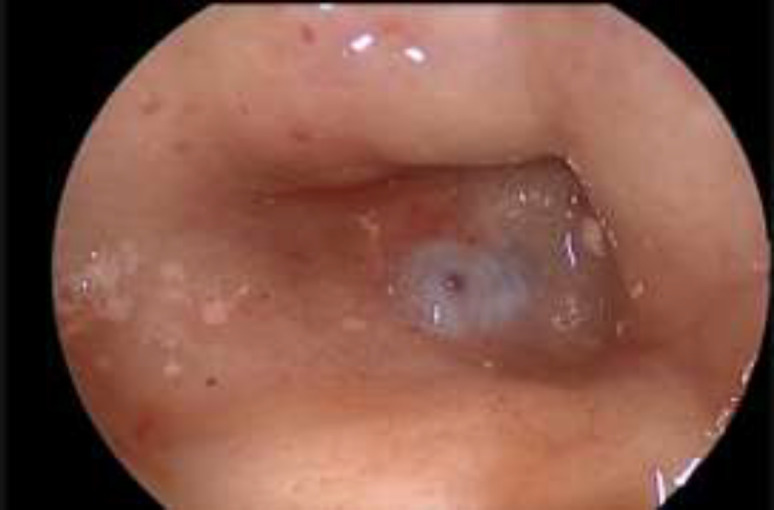
Fistula of the sella

9 out of 10 post-traumatic cases had a leak in in the border area between the anterior and posterior portion of the ethmoid, while 1 patient out of 10 post-traumatic cases had a leak at the level of the sella (Fig. 1).

**Table 1:** The table shows the various types of treated CSF leak

**Number**	**Type**	**Percentage**	**Number**	**Type**	**Percentage**
7 (Total Of 20)	Sfenoid Leak	35% (Total Of 20)	13 (Total Of 20)	Etmoid Leak	65% (Total Of 20)
6	Iatrogenic	30%	4	Iatrogenic	20%
1	Post-Trumatich	5%	9	Post-Traumatic	45%

**Fig 2:**
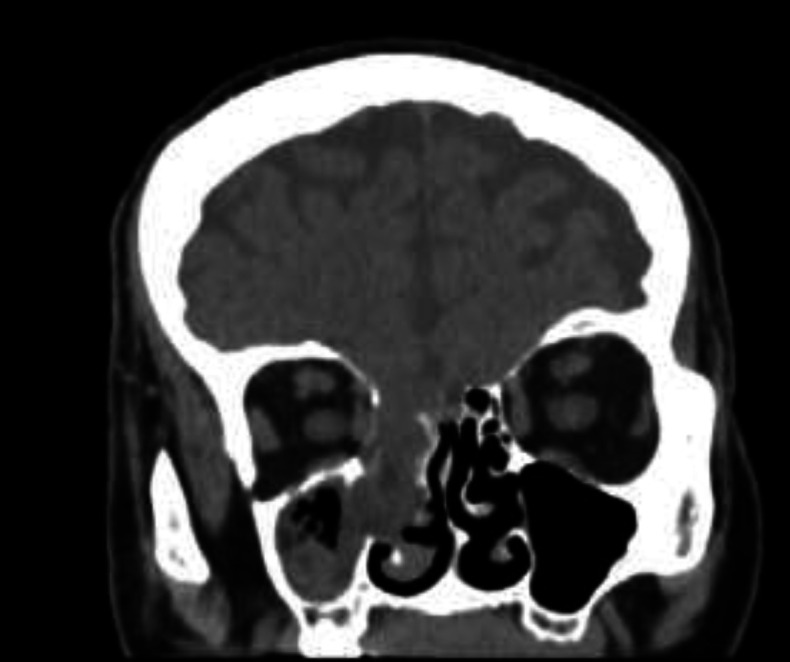
Meningoencephalocele pre-op CT

6 patients out 10 iatrogenic cases presented CSF-leak of the sella after endoscopic removal of pituitary adenoma, while in 4 patients out of 10 iatrogenic cases the CSF-leaks after removal of meningoencephalocele were located between the anterior and posterior ethmoid (Fig. 2). 

**Fig 3:**
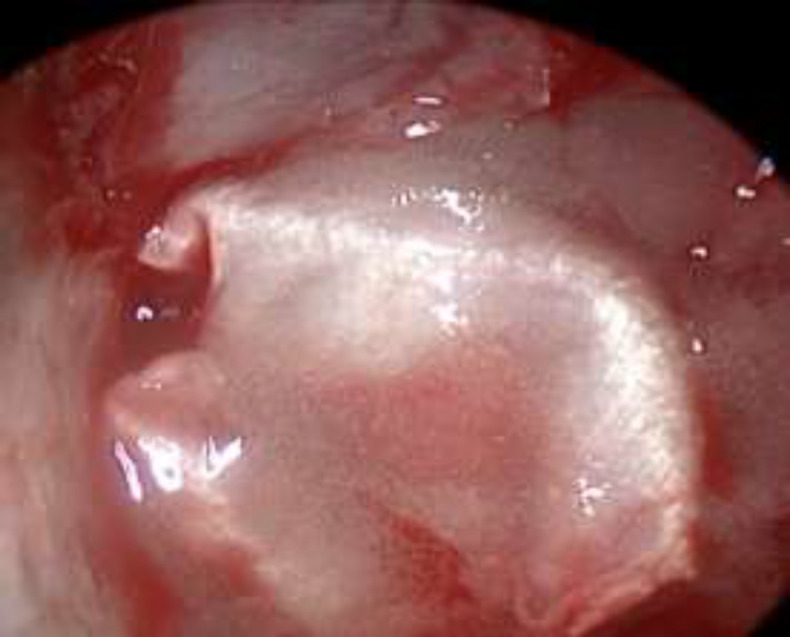
Fascia lata and Vivostat

There were 13 cases of CSF leaks with defects with diameters greater than 1,5 cm and involving anterior and posterior ethmoid therefore in these cases we preferred a triple-layer fascia lata (intracranial intradural, intracranial extradural, extracranial) with Vivostat^® ^interposition and final closure with Hadad flap with adipose tissue and Vivostat^® ^interposition, used as glue and sealant (Fig. 3). 

**Fig 4:**
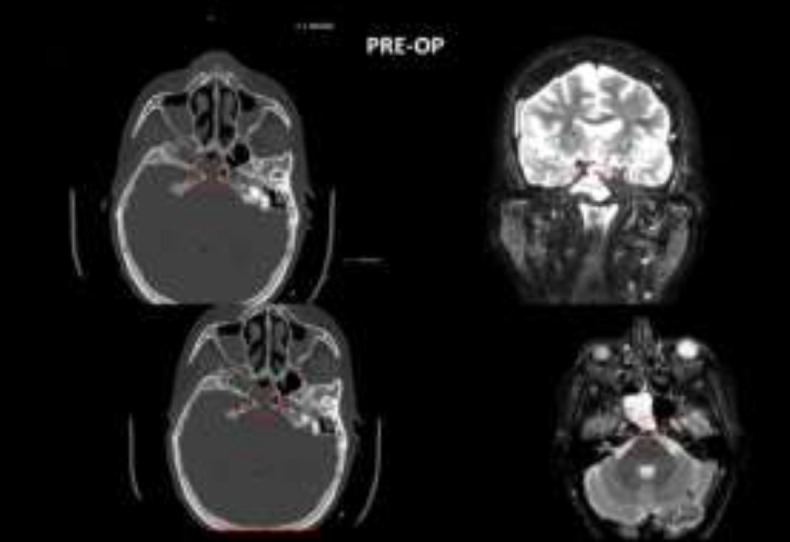
Clivus Fistula pre-op CT

**Fig 5:**
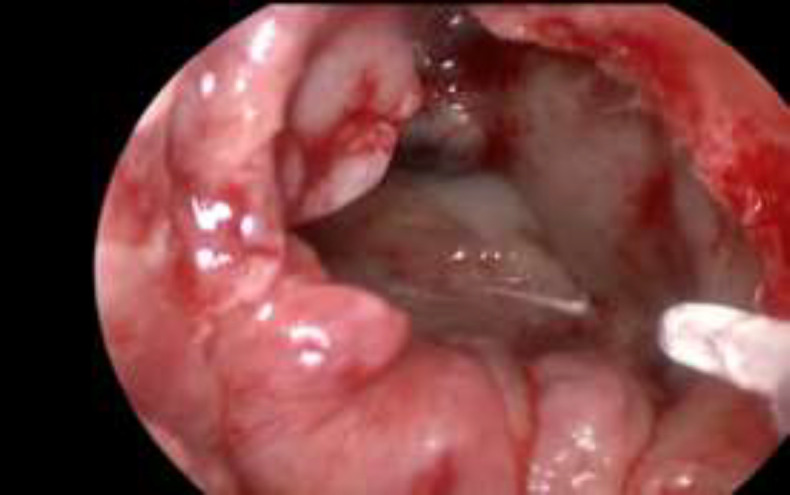
Sfenoid Fat Plug and Vivostat

**Fig 6:**
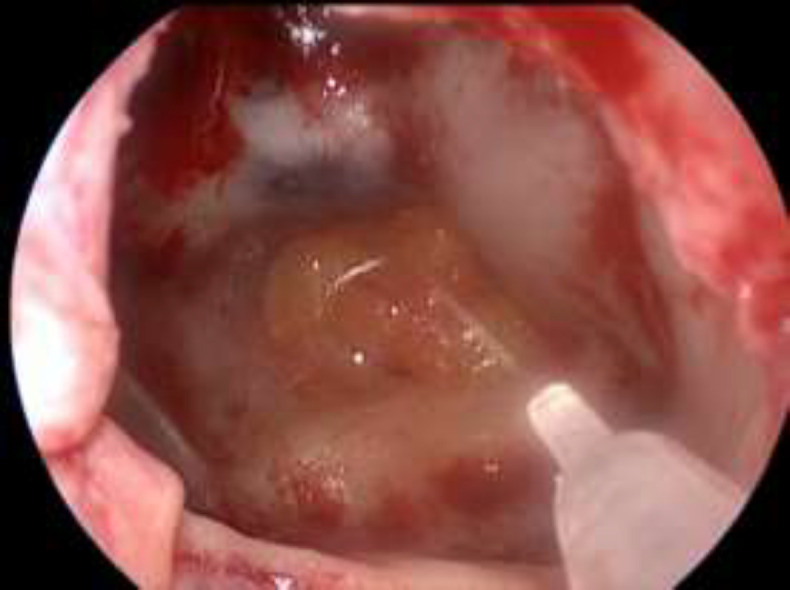
HBF and Vivostat

In leaks of the sphenoid (Fig. 4) inferior to the cm we used Vivostat^®^ interposition with the fat plug technique and overlay closure with Hadad-Bassagasteguy flap (HBF) (Fig. 5-6). In the sphenoid closures after removal of pituitary adenoma we repaired with the fat plug technique and HBF in overlay. All patients were finally buffered with Merocel nasal packing.

**Fig 7:**
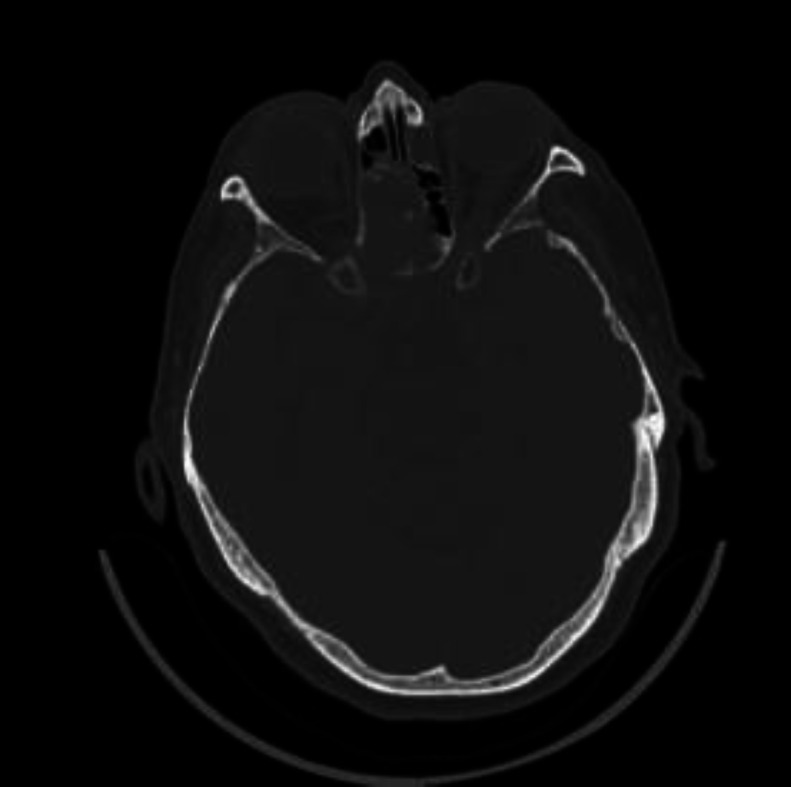
Meningoencephalocele post-op CT

**Fig 8:**
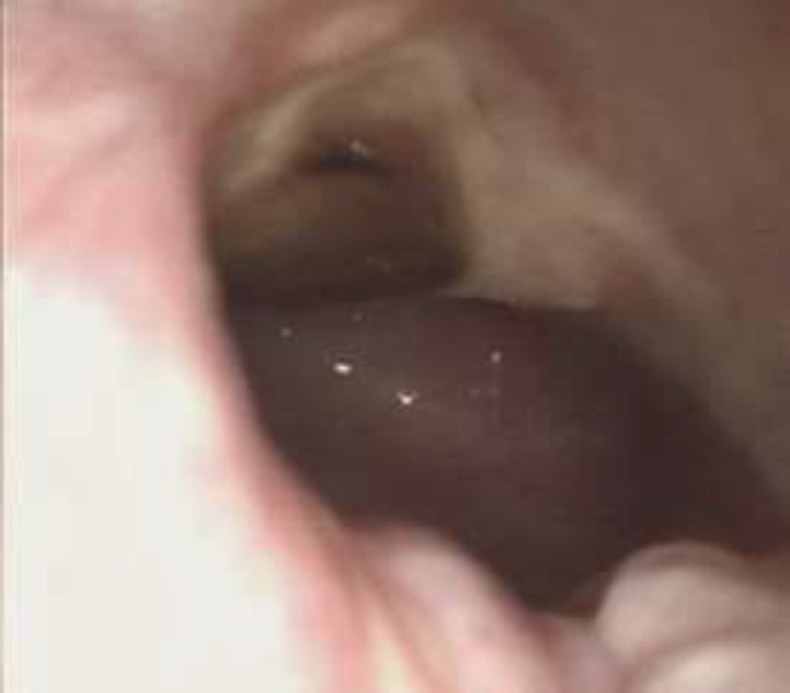
One month control

After 48 h from nasal packing removing no evidence of CSK leaks was observed. CT scan control after 48h didn’t show any CSF leaks in all 20 patients. During this time, we have not registered any kind of complications, or allergic reactions. In 19 patients patients CT scan and endoscopy after 1 month didn’t show any signs of CSF leak (Fig.7,8). 

In one female patient (5% of all patients) a CSF leak between the anterior and posterior ethmoids remained, so a second surgical closure using fluorescein was performed to better identify the leak. In this same case, the use of intraoperative fluorescein revealed the leakage of a small amount of CSF at the level of the previous closure, therefore we choose for a fat-plug closure technique with use of Vivostat^®^ autologous sealant and final closure with mucosa of middle turbinate. All patients reported complete resuscitation of the surgical site after one month, without post-operative periodic haemorrhagic events. The choice to use the Vivostat^®^ system was dictated by its safety for the patient as an autologous derivative and by the possibility of a controlled dosage during its application in the operating field. We have not registered any kind of complications or allergic reactions and our results were satisfactory. 

## Discussion

An anterior skull base CSF leak is usually repaired using the transnasal approach. In 1952 Hirsch was the first to perform a transnasal approach to the sphenoid sinus (1). 

Transnasal approach was later applied by Vrabec and Hallberg to repair a cribriform defect (2). Transnasal was a very revolutionary approach because it avoids an external incision like in the past (transcranial, naso-orbital). In 1981 Wigand described transnasal approach in repairing an anterior skull base defect (3). In 1989 Papay was the first who used an endoscopic endonasal surgery for a skull base CSF leak (4). Kennedy et. al described a minimally invasive technique for repair of CSF rhinorrhea (5,6). Currently the endoscopic technique is the most widespread choice as it allows a correct visualization of the anterior skull bases, the sphenodial sinus (including the lateral portion and the pterygomaxillary space through the use of angled optics) and the frontal sinus. (7,8). The HBF introduction allowed to extend the transnasal approach for its versatile use in closure opening a new era in advanced skull base surgery (9). 

Fibrin glue in skull base surgery is usually used for fixing grafting materials and for its haemostatic function. Conventional fibrin glue is composed of bovine or human borne fibrinogen and thrombin. Some cases of infection by fibrin sealant like Parvovirus B19, other viral transmission and anaphylactic reactions were described in the literature (10-13). Vivostat® autologous system is a safe for patients because is derived from a pre-operative blood sample taken from the patient (14). 

The different type of application pinpointed or sprayed uniformly, the rapid application and density of Vivostat^®^ gives a better grafting material positioning (15). The considerable density allows the Vivostat^® ^to be not washed away by the liquor as easily as normal glues (16,17). In our experience we decided to close CSF leaks with diameters greater than 1,5 cm using a triple fascia layer combined in over and underlayer technique with final HBF. A CSK leak under 1 cm of diameter was repaired with the fat plug technique and HBF.

The Vivostat^®^ system is used for fixing grafting material and for haemostasis. In accordance with Tomazic we agree that Vivostat cannot replace a proper reconstruction technique (15), but it is a very useful adjuvant in fixing and grafting materials (18,19). We also believe that the viscosity of the product is suitable and favors surgical maneuvers during the application of the grafted materials, since the grafts are then positioned in a fairly fluid manner and with the right resistance during application cycles. The ease of use of the endoscopic applicator and the speed of preparation make its use particularly functional in endoscopy surgery. In our experience the use of Vivostat^®^ did not involve any episodes of allergy or infection, so the cost of the product is greatly justified for the sake of greater safety for the patient and the surgeon. We can also point out the fact that the effectiveness of each closure depends only on the proper closure technique, the use of adhesives can never be substitutive to the closing technique, but is nevertheless an important help. In skull base reconstruction is important choosing the right technique independent of the type of sealant used, because the anathomic characteristics of the reconstruction area is different so the technique should be similar to reproduce the original structure. In addition, the points subjected to greater pressure by a gravitational point of view must be taken into consideration, since in certain areas of the skull base, above all in the anterior region, the weight of the brain structures exerts greater pressure, consequently the reconstruction requires greater resistance. 

## Conclusion

Vivostat® is a very safe and useful sealant in endoscopic surgery, avoiding possible complications for patients and improving the surgical reconstructions of the skull bases thanks to both elasticity and resistance. In particular its immediate polimerization is very useful for an instant evaluation of the mechanical seal in the closure of the skull base CSF leaks. The rapid polymerization and the density of Vivostat^® ^are very useful in grafting material positioning. We also consider the costs of the product to be sustainable and justified because of the high level of safety in terms of biocompatibility and reliability for the patient. We also believe that regardless of the safety and effectiveness of the haemostatic used, it is important to choose the correct technique for reconstruction, adapting the various techniques to the type of fistula. To date no isolated product can be used for a correct skull base reconstruction.
